# GMRVGG: A Bearing Fault Diagnosis Method Based on Tri-Modal Image Feature Fusion

**DOI:** 10.3390/s26082426

**Published:** 2026-04-15

**Authors:** Ao Li, Yuantao Li, Xiaoli Wang, Jiancheng Yin

**Affiliations:** School of Mechanical Engineering, Shandong University of Technology, Zibo 255000, China; 24501040035@stumail.sdut.edu.cn (A.L.); 24401020028@stumail.sdut.edu.cn (Y.L.); 24501040032@stumail.sdut.edu.cn (X.W.)

**Keywords:** bearing fault diagnosis, tri-modal image feature fusion, signal-to-image conversion, Gramian Angular Difference Field, Markov Transition Field, Recurrence Plot, VGG16

## Abstract

**Highlights:**

**What are the main findings?**

- A novel bearing fault diagnosis method (GMRVGG) is proposed, which fuses tri-modal features extracted by VGG16 from 1D vibration signals converted into 2D images via GADF, MTF, and RP techniques.
- The proposed methodology demonstrates exceptional robustness, achieving superior overall accuracies on the CWRU and Ottawa datasets, respectively, even under severe 6 dB noise interference.

**What are the implications of the main findings?**

- By integrating diverse signal-to-image conversion techniques, this approach effectively overcomes the limitations of insufficient information dimensionality and inadequate feature representation inherent in conventional single-modal methods.
- It establishes a highly reliable and highly generalizable solution for intelligent condition monitoring and fault diagnosis of rotating machinery operating in complex, noisy industrial environments.

**Abstract:**

Bearings serve as vital components in rotating machinery. Fault diagnosis of bearings constitutes an essential area within mechanical health monitoring. However, most existing methods rely solely on single-modal data or employ a single signal-to-image conversion technique, leading to insufficient information dimensionality and inadequate feature representation, which ultimately limits diagnostic accuracy. To address these challenges, this paper proposes a bearing fault diagnosis method (GADF-MTF-RP-VGG16, GMRVGG) based on tri-modal image feature fusion. Specifically, three image conversion techniques—Gramian Angular Difference Field (GADF), Markov Transition Field (MTF), and Recurrence Plot (RP)—are utilized to first convert 1D vibration signals into 2D images. Subsequently, shallow to deep features are extracted and fused through the VGG16 backbone network. Finally, fault diagnosis is achieved by integrating a fully connected classifier layer. The proposed methodology was comprehensively validated on both the Case Western Reserve University (CWRU) and the University of Ottawa datasets, which were augmented with severe 6 dB Gaussian white noise and 6 dB pink noise to simulate complex industrial environments. Under these harsh conditions, the proposed method achieved superior overall accuracies (up to 96.9% on the CWRU dataset and consistently 95.8% on the Ottawa dataset), significantly surpassing conventional single-modal approaches. This effectively addresses the limitations of insufficient feature dimensionality and inadequate representation, establishing a highly reliable and robust solution for intelligent bearing fault diagnosis.

## 1. Introduction

Modern industry relies heavily on diverse mechanical equipment, wherein bearings invariably play a key role. Functionally, bearings serve as one of the core components in rotating machinery [1], finding critical applications across mechanical manufacturing [2], aerospace [3], wind power generation [4], agriculture [5], and beyond. However, rolling bearings are simultaneously among the most failure-prone parts in modern mechanical systems. For instance, statistics indicate that approximately 40% of motor failures are attributed to bearing failures [6]. Thus, bearing fault diagnosis constitutes an essential aspect of mechanical health monitoring.

In the field of bearing fault diagnosis, while traditional signal processing methods are effective for analyzing individual samples, they often rely on expert knowledge for manual interpretation. Consequently, these methods face challenges in scaling to the massive datasets typical of modern industrial monitoring. Deep learning can uncover latent features from abstract data. With the advancement of artificial intelligence technologies, particularly deep learning techniques, diverse intelligent algorithms have proliferated, thereby furnishing a robust theoretical foundation for intelligent fault diagnosis in bearings [7]. Currently, deep learning has become an essential tool for intelligent bearing fault diagnosis [8]. Many scholars are currently focusing on utilizing deep learning technologies, such as Convolutional Neural Networks (CNN), Recurrent Neural Networks (RNN), and Residual Networks (ResNet), to process vibration signals for bearing fault diagnosis. Cui et al. [9] proposed a bearing fault diagnosis method integrating an attention mechanism with a bidirectional temporal convolutional network (BiTCN). This approach employs SENet to screen fault-related features, utilizes BiTCN to bidirectionally extract temporal dependencies, dynamically weights key features through multi-head attention, and ultimately performs classification via a fully connected layer. On the Jiangnan University and Case Western Reserve University datasets, the method achieved accuracies of 99.49% and 99%, respectively, with an F1-score of 0.98, significantly outperforming comparative models and demonstrating strong generalization capability. Wang et al. [10] designed a 1D Vision ConvNet by parallelizing multi-layer small convolutional kernels and a single-layer large convolutional kernel, validating its effectiveness on both the Case Western Reserve University dataset and the Paderborn University dataset. Si et al. [11] proposed a Multi-Scale Dilated Convolution and Dense Temporal Convolutional Network (MSDC-DenseTCN) model for bearing fault diagnosis under heavy noise conditions. The model first employs a Multi-Scale Dilated Convolution (MSDC) module with three branches of different dilation rates to filter noise and extract multi-scale features, thereby enhancing the receptive field. Subsequently, it utilizes a Dense Temporal Convolutional Network (DenseTCN) module, which combines the feature reuse mechanism of DenseNet and the temporal modeling capability of TCN, to capture long-range temporal dependencies and optimize feature propagation. The model was validated on the Paderborn University and Case Western Reserve University bearing datasets, demonstrating excellent diagnostic performance under various noise conditions. Duan et al. [12] proposed LSBT-Net, a lightweight and interpretable spatiotemporal model, for insulated bearing fault diagnosis. The model first replaces the standard linear projection layer with a multi-scale convolutional architecture to extract both local fine-grained and global coarse-grained features from vibration signals in parallel. It then integrates high-dimensional fault features through a spatiotemporal information fusion module and employs adaptive average pooling to compress spatial dimensions, significantly reducing parameter count and computational resource requirements. Evaluated on wind turbine and rail transit motor insulated bearing datasets, the framework achieved an accuracy of 99.6%. However, despite the demonstrated effectiveness of these deep learning models in processing 1D vibration signals, their performance remains constrained by the inherent limitations of the original time-series data. Specifically, 1D vibration signals often contain substantial non-stationary components and background noise, thereby making it difficult for the models to comprehensively capture the subtle differences and dynamic evolution patterns of fault characteristics. This incompleteness in feature extraction frequently leads to a degradation in model performance.

Image data exhibits superior representational capacity. By employing signal-to-image transformation techniques to convert 1D signals into 2D images, the spatial structures and textural features of the resulting images can be leveraged to clearly reveal the information embedded within the signals. Wang et al. [13] proposed two methods for converting time-series signals into images: the Gramian Angular Field (GAF) based on polar coordinate mapping, and the Markov Transition Field (MTF) based on bin discretization and state transition probability matrices. By employing a tiled convolutional neural network integrated with a linear support vector machine for classifying images generated by these two methods, competitive classification accuracy was achieved on 12 UCR datasets. Daubechies et al. [14] proposed the Synchrosqueezed Wavelet Transform (SST), which generates an initial time-frequency representation through wavelet transform and then compresses it into a high-resolution time-frequency image via reassignment techniques. Its high-precision capability in extracting instantaneous frequencies under strong noise conditions was validated using medical ECG data. Owing to the advantages of image data, numerous scholars in the field of bearing fault diagnosis opt to first convert time-series vibration signals into images, and subsequently extract features contained within these images to accomplish fault diagnosis. Li et al. [15] employed a modified successive variational mode decomposition (SVMD) to extract key signal components. The reconstructed signal was then converted into images via the Gramian Angular Difference Field (GADF) technique and fed into a convolutional neural network for fault classification, achieving an accuracy of 95.2% on the Case Western Reserve University dataset. Wang et al. [16] converted time-series vibration signals into images using the Markov Transition Field (MTF) and input them into a convolutional neural network for bearing fault diagnosis, achieving an accuracy of nearly 100% on the Case Western Reserve University dataset.

However, the aforementioned methods generally suffer from the following limitations: Relying solely on single-modal data for bearing fault diagnosis results in insufficient information dimensionality. Similarly, employing a single signal-to-image conversion technique to process temporal signals leads to inadequate feature representation, as any individual conversion method may fail to comprehensively capture the full characteristics of faults.

To address these limitations, a bearing fault diagnosis method named GADF-MTF-RP-VGG16 (GMRVGG), based on tri-modal image feature fusion, is proposed: The proposed methodology first converts 1D vibration signals into images via three distinct signal-to-image encoding techniques—Gramian Angular Difference Field (GADF), Markov Transition Field (MTF), and Recurrence Plot (RP)—to extract comprehensive signal information. Subsequently, VGG16 is employed to extract features spanning shallow to deep layers from these images. Finally, the feature vectors from different modalities are fused through concatenation and fed into a fully connected classifier for fault diagnosis.

The remainder of this paper is structured as follows: Section 2 provides a detailed introduction to the background methods employed in this study; Section 3 elaborates on the training pipeline and architecture of the GMRVGG framework; Section 4 describes the dataset, experimental methodology, and results; and Section 5 concludes with a summary of the work presented in this paper.

## 2. Background Methods

### 2.1. Gramian Angular Difference Field

The Gramian Angular Difference Field (GADF) is a technique that converts 1D vibration signals into 2D images by characterizing the dynamic evolution of time series through angular differences between time points [17]. This method has been widely applied in fields such as rotating machinery fault diagnosis [18], transmission line fault diagnosis [19], and spectroscopic analysis [20]. During the conversion of 1D vibration signals into images, the GADF preserves the critical information within the time series [21]. Fluctuations in 1D vibration signals are mapped into cross-shaped patterns within their corresponding GADF images. The greater the amplitude of these fluctuations, the more pronounced the cross-shaped patterns become in the imagery. Therefore, GADF images can effectively capture fault impulses within bearing vibration signals and characterize the fault features present during the vibration process [22]. For a given time series $X=\left\{x_{1},x_{2},\cdots ,x_{n}\right\}$, where n denotes the length of the time series sequence and $x_{i}$ represents the vibration signal amplitude at the i-th time step, the GADF transformation is defined as follows:

(1) Normalization: For a given time series $X=\left\{x_{1},x_{2},\cdots ,x_{n}\right\}$, it is normalized to the range $\left[-1,1\right]$. Let $\tilde{X}=\left\{{\tilde{x}}_{1},{\tilde{x}}_{2},\cdots ,{\tilde{x}}_{n}\right\}$ denote the normalized time series.

(1) $${\tilde{x}}_{i}\begin{array}{c} =\frac{\left(x_{i}-\max\left(X\right)\right)+\left(x_{i}-\min\left(X\right)\right)}{\max\left(X\right)-\min\left(X\right)},x_{i}\in X \end{array},$$

where $x_{i}$ is the raw signal and ${\tilde{x}}_{i}$ denotes the normalized signal.

(2) Convert to polar coordinates.

(2) $$\left\{\begin{array}{c} \phi =\arccos\left({\tilde{x}}_{i}\right),-1\leq {\tilde{x}}_{i}\leq 1,{\tilde{x}}_{i}\in \tilde{X}\\ r=\frac{t_{i}}{N},t_{i}\in N \end{array}\right.,$$

where $t_{i}$ denotes the time index, and N represents a constant scaling factor controlling the range of polar coordinates.

(3) Constructing the Gramian Angular Difference Field.

(3) $$GADF=\left[\begin{array}{ccc} sin(\phi_{1}-\phi_{1}) & \cdots & sin(\phi_{1}-\phi_{n})\\ sin(\phi_{2}-\phi_{1}) & \cdots & sin(\phi_{2}-\phi_{n})\\ \vdots & \ddots & \vdots \\ sin(\phi_{n}-\phi_{1}) & \cdots & sin(\phi_{n}-\phi_{n}) \end{array}\right],$$

**Figure 1:**
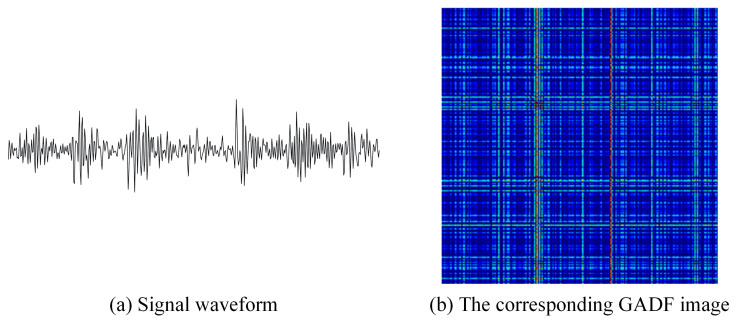
Illustration of the GADF transformation process. (**a**) Raw vibration signal waveform under an outer race fault condition. (**b**) The corresponding converted GADF image. The colors in the image correspond to the scalar values mapped by the “jet” colormap, indicating the trigonometric difference between angular points.

Taking a segment of signal under outer race fault condition as an example, it is converted into a 2D image using the GADF and “jet” colormap. The generated GADF image is shown in Figure 1.

As shown in Figure 1, the 1D vibration signal is mapped into a two-dimensional matrix based on the trigonometric difference between angular points, forming a cross-like texture pattern.

### 2.2. Markov Transition Field

The Markov Transition Field (MTF) is a technique that converts 1D vibration signals into 2D images by leveraging the dynamic evolution process of temporal signals [23]. It has been widely applied in fields such as fault diagnosis in rotating machinery [24] and condition monitoring of industrial equipment [25]. The MTF constructs a Markov transition matrix through the state transition probabilities of adjacent temporal nodes, which is then expanded into a transition field and ultimately mapped to a grayscale image via downsampling. When processing raw time-series vibration signals directly with CNNs, potential information loss may occur. In contrast, the MTF effectively preserves the temporal characteristics of the signals, thereby enhancing the capability of CNNs in handling time-series data [26]. For a given time series $X=\left\{x_{1},x_{2},\cdots ,x_{n}\right\}$, where n denotes the length of the time series sequence and $x_{i}$ represents the vibration signal amplitude at the i-th time step, the MTF transformation is defined as follows:

(1) Discretization: For a given time series $X=\left\{x_{1},x_{2},\cdots {,x}_{n}\right\}$, partition the time series values into Q quantile bins, with each $x_{i}$ mapped to its corresponding quantile $q_{j}\left(j\in \left[1,Q\right]\right)$.
(2) Constructing the Markov transition matrix:

The transition probability $w_{ij}$ from quantile bin $q_{i}$ to $q_{j}$ is defined as:

(4) $$w_{ij}=p\left\{x_{t+1}\in q_{j}|x_{t}\in q_{i}\right\},$$

where $x_{t}$ represents the signal amplitude at time t, and $q_{i}$ indicates the quantile region corresponding to $x_{i}$.

Specifically, $w_{ij}$ is computed by counting the transition frequency $N_{ij}$ from bin $q_{i}$ to bin $q_{j}$ and performing row normalization:

(5) $$w_{ij}=\frac{N_{ij}}{\sum_{k=1}^{Q}N_{ik}},$$

where $N_{ij}$ denotes the number of times a point in bin $q_{i}$ is immediately followed by a point in bin $q_{j}$.

This results in the Markov Transition Matrix W.

(6) $$W=\left[\begin{array}{cccc} w_{11} & w_{12} & \cdots & w_{1Q}\\ w_{21} & w_{22} & \cdots & w_{2Q}\\ \vdots & \vdots & \ddots & \vdots \\ w_{Q1} & w_{Q2} & \cdots & w_{QQ} \end{array}\right],$$

(3) Expanded into the Markov Transition Field.

(7) $$MTF=\left[\begin{array}{ccc} w_{ij}|x_{1}\in q_{i},x_{1}\in q_{j} & \cdots & w_{ij}|x_{1}\in q_{i},x_{n}\in q_{j}\\ w_{ij}|x_{2}\in q_{i},x_{1}\in q_{j} & \cdots & w_{ij}|x_{2}\in q_{i},x_{n}\in q_{j}\\ \vdots & \ddots & \vdots \\ w_{ij}|x_{n}\in q_{i},x_{1}\in q_{j} & \cdots & w_{ij}|x_{n}\in q_{i},x_{n}\in q_{j} \end{array}\right],$$

**Figure 2:**
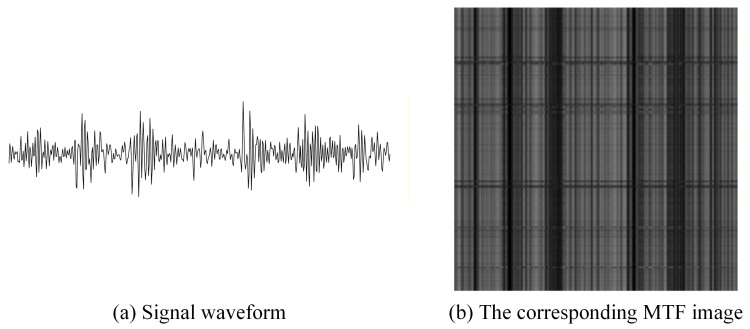
Illustration of the MTF transformation process. (**a**) Raw vibration signal waveform under an outer race fault condition. (**b**) The corresponding converted MTF image. The grayscale intensities in the image represent the transition probabilities between different quantile bins.

Taking a segment of signal under outer race fault condition as an example, it is converted into a 2D image using the MTF. The generated MTF image is shown in Figure 2.

As shown in Figure 2, the pixel intensities in the image represent the Markov transition probabilities between different quantile bins of the time-series data.

### 2.3. Recurrence Plot

The Recurrence Plot (RP) employs phase space reconstruction technology to generate 2D images by quantifying the recurrence characteristics between state points in dynamic systems [27]. This technique enables the visual conversion of 1D signals into high-dimensional dynamic features, and it has been widely applied in fields such as fault diagnosis for rotating machinery [28] and fluid dynamics research [29]. The RP algorithm is capable of effectively detecting characteristic patterns hidden within short-term non-smooth time series, making it well-suited for bearing fault diagnosis scenarios [30]. For a given time series $X=\left\{x_{1},x_{2},\cdots ,x_{n}\right\}$, where n denotes the length of the time series sequence and $x_{i}$ represents the vibration signal amplitude at the i-th time step, the RP transformation is defined as follows:

(1) Phase Space Reconstruction: For a given time series $X=\left\{x_{1},x_{2},\cdots {,x}_{n}\right\}$, construct the phase space vectors.

(8) $${\vec{v}}_{i}=\left(x_{i},x_{i+\tau },x_{i+2\tau },\cdots ,x_{i+(m-1)\tau }\right),i=1,2,3\cdots N,N=n-\left(m-1\right)\tau ,$$

where m denotes the embedding dimension and τ represents the time delay.

(2) Computing the Distance Matrix (Using Euclidean Distance as an Example).

(9) $$\begin{array}{c} D=\left[\begin{array}{cccc} d_{11} & d_{12} & \cdots & d_{1N}\\ d_{21} & d_{22} & \cdots & d_{2N}\\ \vdots & \vdots & \ddots & \vdots \\ d_{N1} & d_{N2} & \cdots & d_{NN} \end{array}\right],d_{ij}=\left\|{\vec{v}}_{i}-{\vec{v}}_{j}\right\|= \end{array}\sqrt{\sum_{k=0}^{m-1}{\left(x_{i+k\tau }-x_{j+k\tau }\right)}^{2}},$$

(3) Transformed into a continuous recurrence matrix via the Gaussian kernel function.

(10) $$\begin{array}{c} RP=\left[\begin{array}{cccc} r_{11} & r_{12} & \cdots & r_{1N}\\ r_{21} & r_{22} & \cdots & r_{2N}\\ \vdots & \vdots & \ddots & \vdots \\ r_{N1} & r_{N2} & \cdots & r_{NN} \end{array}\right],r_{ij}=\exp\left(-\frac{d_{ij}^{2}}{2\sigma^{2}}\right) \end{array}$$

where σ is the Gaussian kernel parameter that controls the sensitivity of recurrence.

**Figure 3:**
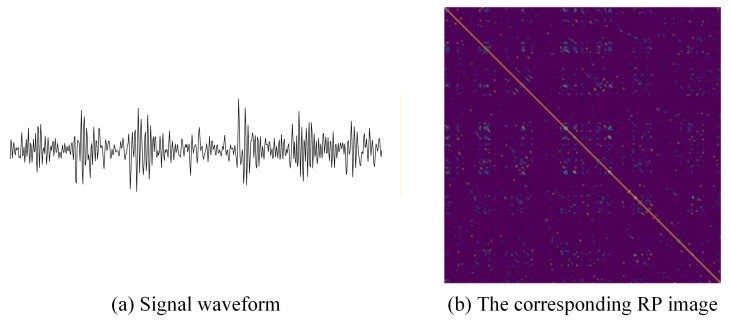
Illustration of the RP transformation process. (**a**) Raw vibration signal waveform under an outer race fault condition. (**b**) The corresponding converted RP image. The colors in the image correspond to the distance values calculated by the Gaussian kernel, visualized using the “viridis” colormap.

Taking a segment of signal under outer race fault condition as an example, it is converted into a 2D image using the RP and “viridis” colormap. The generated RP image is shown in Figure 3.

As shown in Figure 3, the image visualizes the recurrence states of the phase space trajectories, where the colors correspond to the distance values calculated by the Gaussian kernel.

### 2.4. Convolutional Neural Network and VGG16

**Figure 4:**
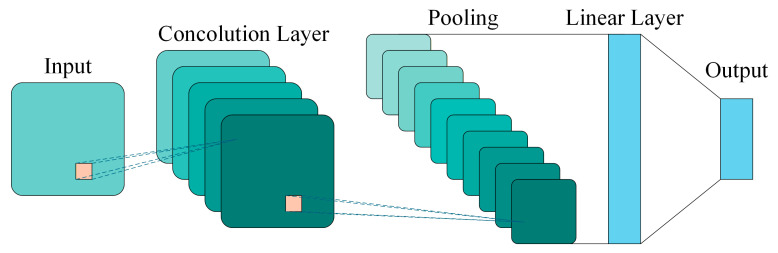
Architecture of the CNN.

Convolutional Neural Networks (CNNs) represent a class of deep learning architectures specifically engineered to process data with grid-like topologies, such as images and time-series signals. The fundamental CNN architecture comprises convolutional layers, pooling layers, and linear layers, enabling the extraction of hierarchical features from images for accurate and efficient recognition and classification [31]. Although CNNs are typically used for end-to-end classification, their intermediate layers contain rich, high-level abstract representations of the input data. Therefore, by extracting the output vectors from the hidden fully connected layers preceding the final output layer, the CNN can be effectively utilized as a deep feature extractor to generate high-dimensional feature representations for downstream tasks. Currently, CNN have been widely applied in fields such as bearing fault diagnosis, becoming one of the core tools in deep learning. The typical architecture of a CNN is shown in Figure 4.

As shown in Figure 4, the architecture of a classical CNN is formed by stacking convolutional layers, pooling layers, and linear layers.

**Figure 5:**
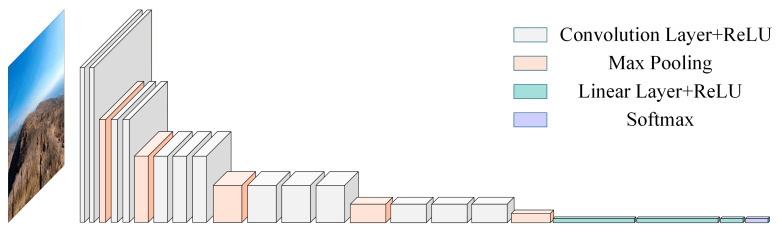
Architecture of the VGG16 network. The input image is a landscape photograph taken personally by the author (Ao Li) at Fuxi Mountain, Zhengzhou, China. The author holds the copyright and grants permission to publish it under the CC BY 4.0 license.

VGG16 is a classic deep convolutional neural network model in the field of deep learning, proposed in 2014 by the Visual Geometry Group at the University of Oxford. VGG16 achieved breakthrough performance in the ImageNet Large Scale Visual Recognition Challenge (ILSVRC), and the model can be effectively applied to other datasets through transfer learning [32]. The architecture of VGG16 is shown in Figure 5.

As shown in Figure 5, the VGG16 network consists of 13 convolutional layers and 3 linear layers. The convolutional layers are organized into 5 blocks, where each block is followed by a max-pooling layer to progressively reduce the spatial dimensions. Small 3 × 3 convolutional filters are employed throughout the network to capture features. Finally, the feature maps are flattened and passed through the linear layers, followed by a Softmax layer to generate the final classification probabilities.

## 3. Proposed Method

### 3.1. Methodology Flow

**Figure 6:**
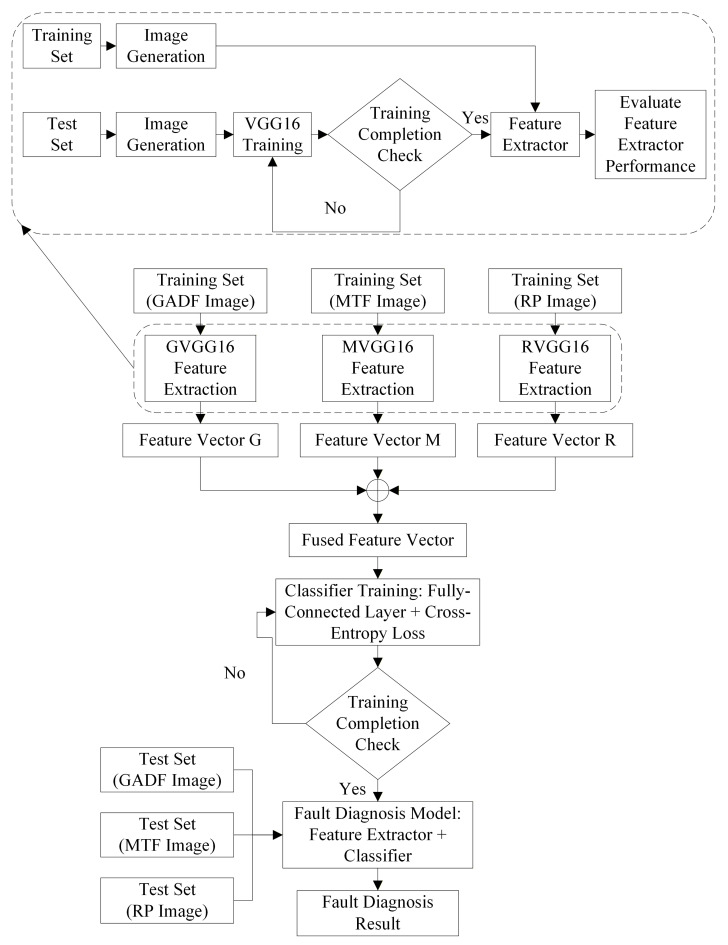
Training pipeline of the GMRVGG framework. The pipeline illustrates the two-stage training process, including the feature extractor training for each modality and the final classifier training.

To address the limitations of insufficient information dimensionality and inadequate feature representation in single-modal models, a novel GMRVGG-based bearing fault diagnosis method is proposed, which integrates three signal-to-image conversion techniques (GADF, MTF, RP) with VGG16 for tri-modal feature fusion. The training of the proposed method consists of two distinct stages: initially training the feature extractor, and subsequently training the fusion classifier after fixing the optimal parameters of the feature extractor. This modular design offers significant flexibility, allowing for straightforward addition, removal, or substitution of various modalities. Crucially, changes to the feature extractor of any single modality do not affect the extractors of other modalities. The training pipeline is illustrated in Figure 6.

As shown in Figure 6, the training pipeline of the GMRVGG Framework is as follows:

Step 1: Train the single-modality feature extractor.

Step 1.1: Preprocess the signals and partition the dataset into training and test sets proportionally.

Step 1.2: Convert the samples in both the training set and test set into their corresponding images using GADF, MTF, and RP, respectively.

GADF transformation: The 1D signals are converted into GADF images based on Equation (1) to (3) in Section 2.1. In this study, to match the input dimension of the VGG16 network, the value of N is determined as 224. To accommodate the input requirements of the VGG16 architecture, the scalar values of the GADF matrices were transformed into the RGB color space using the “jet” colormap.

MTF transformation: The 1D signals are converted into MTF images based on Equation (4) to (7) in Section 2.2.

RP transformation: The 1D signals are converted into RP images based on Equation (8) to (10) in Section 2.3. In this study, the parameters were set as embedding dimension m=2 and time delay τ=1. Consequently, the reconstructed vector simplifies to $\begin{array}{c} {\vec{v}}_{i}=\left(x_{i},x_{i+1}\right) \end{array}$. This configuration preserves the complete dynamic evolution patterns information of the vibrational signals, avoiding potential information loss caused by large time delays, thereby providing rich input features for the subsequent VGG16 network. To accommodate the input requirements of the VGG16 architecture, the Gaussian-kernelized recurrence matrices were visualized using the “viridis” colormap.

Step 1.3: Input the training set images from the GADF branch into the VGG16 network for training. The network parameters are iteratively updated via the backpropagation algorithm using the Adam optimizer to minimize the Cross-Entropy Loss. The weights corresponding to the highest classification accuracy on the validation set are preserved as the optimal weights for the branch.

The Cross-Entropy loss function is a highly suitable choice for multi-class classification tasks. It effectively quantifies the disparity between the predicted probability distribution and the true distribution of the labels. Compared to other loss functions, such as the Mean Squared Error, the Cross-Entropy loss generates stronger gradients for incorrect predictions, thereby accelerating the convergence of gradient descent algorithms [33]. The Cross-Entropy Loss function is defined as follows:

(11) $$\begin{array}{c} L=-\frac{1}{N}\sum_{i=1}^{N}\sum_{c=1}^{C}y_{c}^{\left(i\right)}log({\hat{y}}_{c}^{\left(i\right)}) \end{array},$$

where N represents the number of samples in a batch; C denotes the total number of classes; $y_{c}^{\left(i\right)}$ is a binary indicator (0 or 1) specifying whether class c is the true classification for sample i; and ${\hat{y}}_{c}^{\left(i\right)}$ is the predicted probability that sample i belongs to class c.

Select the Rectified Linear Unit (ReLU) activation function. The ReLU activation function is defined as follows:

(12) $$f\left(x\right)=\left\{\begin{array}{c} x,\;\;if\;x>0\\ 0,\;\;if\;x\leq 0 \end{array}\right.$$

where x is the input to the nonlinear activation function f across all input feature channels.

In this study, the variable m is used to represent the minibatch size. Considering the high memory consumption of the VGG16 architecture, the minibatch size was set to m=4 for this feature extractor training phase.

Step 1.4: Load the optimal weights and input the test set images into the trained VGG16 for testing.

Step 1.5: Upon completion of training and testing, fix the weights of the feature extractor, referred to as GVGG16. Specifically, the output of the first fully connected layer is extracted as the feature vector for the subsequent fusion process.

Step 1.6: The same procedure is applied to train and test the feature extractors for the MTF and RP branches, resulting in the MVGG16 and RVGG16 feature extractors with fixed weights after training.

Step 2: Train the final fully connected classifier.

Step 2.1: Extract feature vectors from the training set images of GADF, MTF, and RP modalities using three branch feature extractors respectively. For each data sample, the proposed model extracts a 4096-dimensional feature vector.

Step 2.2: Concatenate the three distinct feature vectors to form a unified 12,288-dimensional vector and feed them into a fully connected classifier. Train the classifier using cross-entropy loss to optimize its parameters, and preserve the optimal weights. Although these features originate from the same signal and may share semantic overlaps, their fusion provides complementary visual perspectives, enabling the classifier to learn a more comprehensive representation. Similarly, the Adam optimizer is employed to update the weights iteratively. The model parameters that achieve the highest classification accuracy on the validation set are saved as the optimal weights for the final fault diagnosis model. For this lightweight fusion classifier, the minibatch size was increased to m=32 to facilitate stable convergence.

Use Batch Normalization (BatchNorm) to accelerate the training process, enhance training stability, and confer a regularization effect. For a mini-batch of data $X=\left\{x_{1},x_{2},\cdots x_{n}\right\}$, the BatchNorm formulation is defined as follows:

(1) Compute the mean and variance of the current mini-batch data.

(13) $$\mu_{X}=\frac{1}{m}\sum_{i=1}^{m}x_{i},$$

(14) $$\sigma_{X}^{2}=\frac{1}{m}\sum_{i=1}^{m}(x_{i}-\mu_{X}{)}^{2},$$

where X denotes the current mini-batch data; m represents the size of the mini-batch; $x_{i}$ indicates the activation value of the i-th sample in the mini-batch; $\mu_{X}$ signifies the mean of the current mini-batch; and $\sigma_{X}^{2}$ denotes the variance of the current mini-batch.

(2) Standardization.

(15) $${\hat{x}}_{i}=\frac{x_{i}-\mu_{X}}{\sqrt{\sigma_{X}^{2}+\epsilon }},$$

where ϵ denotes a small constant to avoid division by zero.

(3) Scaling and Shifting.

(16) $$y_{i}=\gamma {\hat{x}}_{i}+\beta ,$$

where γ denotes the scale factor; β represents the shift factor. Both γ and β are learnable parameters.

Dropout is a regularization technique designed to prevent overfitting [34]. The formulation of Dropout is defined as follows:

(17) $$\tilde{x}=\frac{1}{1-p}m⊙x,$$

where m denotes the binary mask, with 0 indicating dropout and 1 indicating retention; p represents the dropout probability.

Step 2.3: Load the optimal weights, process the test set images through the three branch feature extractors and the classifier for final testing and evaluation, and complete the multimodal bearing fault diagnosis task.

**Figure 7:**
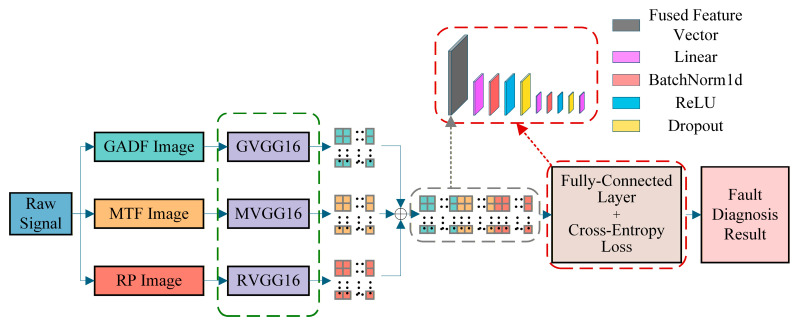
Architecture of the GMRVGG. The model integrates three branches (GADF, MTF, RP) with VGG16 backbones and fuses features via concatenation before the final classification. The arrays of colored squares interspersed with dots denote the extracted high-dimensional feature vectors corresponding to each modality (cyan for GADF, orange for MTF, and red for RP). The expanded dashed box illustrates the detailed internal layer sequence of the fully connected classifier.

The architecture of the proposed model is illustrated in Figure 7.

As shown in Figure 7, the overall architecture of the GMRVGG model consists of three parallel VGG16 feature extraction branches, which extract high-dimensional features from GADF, MTF, and RP images, respectively. These multi-modal feature vectors are fused via concatenation and then fed into a fully connected classifier to output the final bearing fault diagnosis results.

### 3.2. Network Parameters

The network parameters of the VGG16 feature extractor are listed in Table 1.

As shown in Table 1, the model accepts an input image of size 224 × 224 × 3. Through a series of convolutional and pooling layers, the spatial resolution is progressively reduced while the depth is increased. Finally, the feature map is flattened and processed by the linear layer to output a 4096-dimensional feature vector, which serves as the input for the subsequent fusion module.

The network parameters of the fully connected classifier are listed in Table 2.

As shown in Table 2, the classifier receives a fused feature vector with a dimensionality of 12,288, formed by concatenating three 4096-dimensional vectors. Its architecture comprises three linear layers. To ensure training stability and prevent overfitting, BatchNorm and Dropout layers are incorporated between the linear layers. The final layer maps the features to four output nodes, corresponding to the number of fault categories.

### 3.3. Implementation Details

To ensure the reproducibility of the proposed method, the hyperparameters used for training are listed in Table 3.

As shown in Table 3, the model was implemented using the PyTorch framework (version 2.6.0). The Adam optimizer was employed with an initial learning rate of 1 × 10^−4^ for all phases. To ensure stable convergence, a StepLR scheduler was used to decay the learning rate.

Regarding data preprocessing, all generated GADF, MTF, and RP images were resized to 224 × 224 pixels. Subsequently, standard ImageNet normalization (mean = [0.485, 0.456, 0.406], std = [0.229, 0.224, 0.225]) was applied to facilitate the convergence of the pre-trained VGG16 backbone.

## 4. Results and Discussion

### 4.1. Experiment I: Diagnostic Performance Analysis on CWRU Dataset

#### 4.1.1. Dataset Introduction

In this section, the bearing fault diagnosis dataset from Case Western Reserve University (CWRU) is employed for experimental validation. The CWRU bearing dataset was developed by the Mechanical Engineering Laboratory at Case Western Reserve University in the United States. The experimental setup utilized a 2 hp Reliance Electric motor (Reliance Electric, Greenville, SC, USA), with acceleration data measured both near and away from the motor bearings [35]. The experiments utilized SKF 6205-2RS JEM deep groove ball bearings (SKF, Gothenburg, Sweden), which are standard industrial metallic bearings.

Bearing faults are induced by Electric Discharge Machining (EDM). The fault signals are categorized into two types: drive-end bearing signals and fan-end bearing signals. The motor operates under four load conditions: 0 hp, 1 hp, 2 hp, and 3 hp. The fault types include ball faults, inner race faults, and outer race faults. The fault diameters are artificially seeded at 0.007 inch, 0.014 inch, 0.021 inch, and 0.028 inch. The bearing fault signals are sampled at two frequencies: 12,000 Hz and 48,000 Hz.

For the experiments in this section, drive-end bearing signals under a 0 hp load condition with a sampling frequency of 12,000 Hz were selected. Fault diameters of 0.007 inch, 0.014 inch, and 0.021 inch were chosen, and combined with signals from the normal state, this resulted in a total of ten distinct categories of bearing fault signal data. Signals of the same fault type but with different fault diameters are grouped under the same label, resulting in four final categories: “Normal”, “Ball”, “IR”, and “OR”.

**Figure 8:**
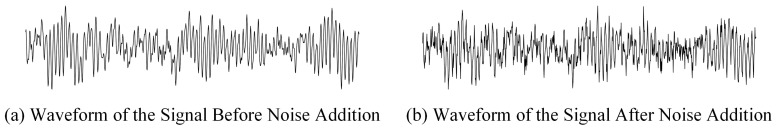
Signal waveform comparison under 6 dB Gaussian white noise. (**a**) Original signal before noise addition. (**b**) Signal after adding 6 dB Gaussian white noise.

In practical industrial settings, vibration signals from bearings are inevitably contaminated by background noise. To rigorously evaluate the robustness of the proposed method against noise interference, artificial noise is introduced into the original signals prior to sample segmentation. Acknowledging that real-world industrial noise is often complex and machine-condition-dependent, this study employs two distinct types of noise for comprehensive validation: Gaussian white noise and pink noise, both at 6 dB. Gaussian white noise is employed to simulate ideal random measurement noise, whereas pink noise is utilized to characterize the low-frequency background noise prevalent in practical industrial environments [36]. A comparison of the signal waveforms before and after adding 6 dB Gaussian white noise is shown in Figure 8.

**Figure 9:**
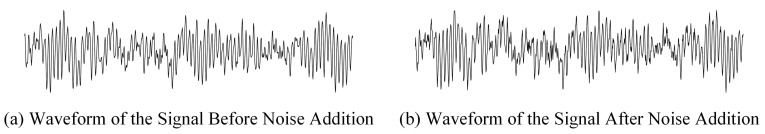
Signal waveform comparison under 6 dB pink noise. (**a**) Original signal before noise addition. (**b**) Signal after adding 6 dB pink noise.

As shown in Figure 8, the waveform of the signal undergoes a significant transformation, characterized by a marked increase in random irregularities and intense fluctuations in the amplitude of its peaks and troughs. This comparison visually illustrates the significant impact of the added noise. A comparison of the signal waveforms before and after adding 6 dB pink noise is shown in Figure 9.

As shown in Figure 9, the waveform also exhibits a significant transformation. In contrast to the sharp irregularities of white noise, this change is characterized by more pronounced low-frequency modulation and a blurring of the distinct peaks and troughs.

To prevent data leakage, for each noise scenario, the data points with added noise were partitioned into training and test sets in an 8:2 ratio. Subsequently, samples were obtained through sequential segmentation with a sample length set to 512 data points and an overlap ratio of 50%. The details of the processed CWRU dataset partition are listed in Table 4.

As shown in Table 4, the dataset is categorized into four classes (Normal, Ball, IR, OR) labeled 0 to 3. To prevent the model from becoming biased toward majority classes and to ensure unbiased evaluation across all fault types, a strictly balanced dataset was constructed. For each noise scenario, each category contains 108 training samples and 24 test samples, ensuring a balanced distribution for model training and evaluation.

#### 4.1.2. Experimental Results

(1) Performance under 6 dB Gaussian white noise

For the method proposed in this paper, the experimental results on the CWRU dataset under the 6 dB Gaussian white noise condition are listed in Table 5.

As can be seen from Table 5, in this noise environment, the proposed method achieves significantly higher accuracy than other single-modal methods. Under the interference of 6 dB Gaussian white noise, single-modal methods can only achieve high accuracy in identifying certain specific fault types, whereas the tri-modal fusion approach is capable of recognizing each fault type with consistently high accuracy. The experimental results demonstrate that the proposed multimodal fault diagnosis model exhibits a significant advantage in accuracy compared to other single-modal diagnostic models. It is important to note that, as the dataset is strictly balanced with an equal number of samples for each class, the variations in classification accuracy are not caused by class imbalance. Instead, they are solely determined by the distinctiveness of the feature representations learned by the model for different fault types.

**Figure 10:**
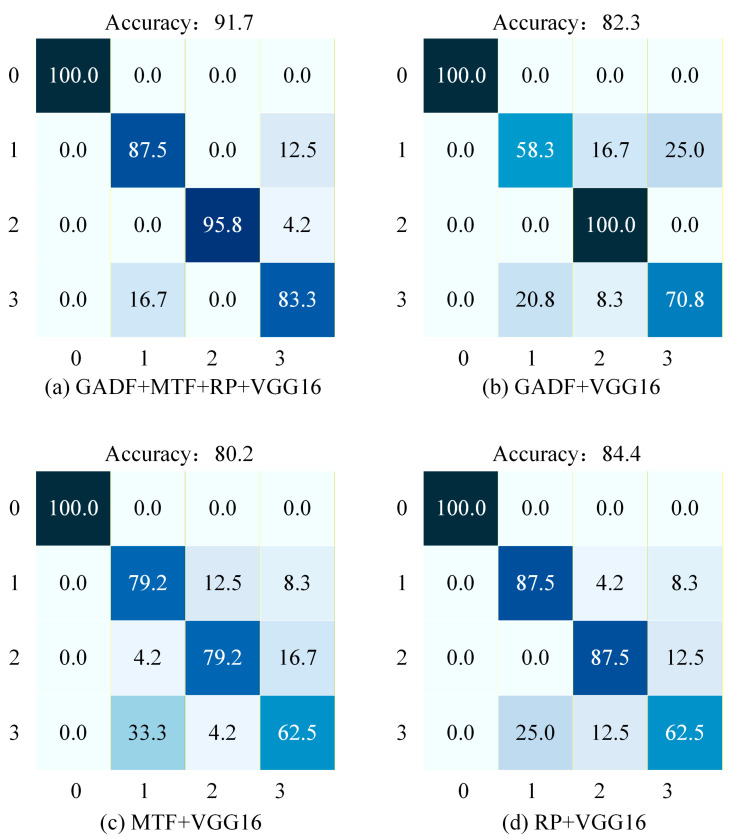
Confusion matrices of the four methods under 6 dB Gaussian white noise. (**a**) Proposed GMRVGG (Tri-modal). (**b**) GADF + VGG16. (**c**) MTF + VGG16. (**d**) RP + VGG16. The color intensity represents the percentage of classification accuracy, with darker shades indicating higher values.

To more intuitively evaluate the performance of the fault diagnosis model, this paper also introduces the confusion matrix. The confusion matrix clearly reveals the relationship between the predicted values and the true values. By comparing the color intensity within the matrix, one can intuitively assess the performance of fault classification. Figure 10 shows the confusion matrices for the four methods under 6 dB Gaussian white noise. In these matrices, the horizontal axis represents the predicted labels, while the vertical axis represents the true labels, where 0 represents the Normal condition, 1 represents the Ball Fault, 2 represents the Inner Race Fault, and 3 represents the Outer Race Fault.

As illustrated in Figure 10a, the proposed GMRVGG16 model achieves an overall accuracy of 91.7% and demonstrates high diagnostic accuracy across all fault categories. Specifically, the classification accuracy for the Normal state reaches 100% with no misclassifications. For Ball Faults, the accuracy is 87.5%, with the primary error being misclassification as Outer Race Faults (12.5%). Inner Race Faults achieve a high accuracy of 95.8%, with minor misclassification as Outer Race Faults (4.2%). The accuracy for Outer Race Faults is 83.3%, where the main confusion occurs with Ball Faults (16.7%). Overall, the GMRVGG16 model maintains high diagnostic accuracy and stability across all fault types.

As illustrated in Figure 10b, the GADF + VGG16 model achieves an overall accuracy of 82.3%, yet exhibits significant fluctuations across different fault categories. Specifically, it attains 100% accuracy in classifying the Normal state, correctly identifying all samples. However, for Ball Faults, the accuracy drops sharply to 58.3%, with primary misclassifications occurring as Inner Race Faults (16.7%) and Outer Race Faults (25.0%). In contrast, Inner Race Faults are perfectly classified with 100.0% accuracy and no errors. For Outer Race Faults, the accuracy is 70.8%, dominated by misclassifications as Ball Faults (20.8%) and Inner Race Faults (8.3%). Overall, this method demonstrates a pronounced deficiency in diagnosing Ball Faults, indicating limited robustness for this specific fault type.

As illustrated in Figure 10c, the MTF + VGG16 model achieves an overall accuracy of 80.2%. The classification accuracy for the Normal state reaches 100% with no misclassifications. The accuracy for Outer Race Faults is the lowest at 62.5%, with primary errors including misclassification as Ball Faults (33.3%) and Inner Race Faults (4.2%). For Ball Faults, the accuracy is 79.2%, with main errors being misclassified as Inner Race Faults (12.5%) and Outer Race Faults (8.3%). The accuracy for Inner Race Faults is also 79.2%, dominated by misclassifications as Ball Faults (4.2%) and Outer Race Faults (16.7%). Overall, this method proves to be particularly weak in diagnosing Outer Race Faults.

As illustrated in Figure 10d, the RP + VGG16 model achieves an overall accuracy of 84.4%, yet its diagnostic results exhibit certain instability. Specifically, it attains 100.0% accuracy in classifying the Normal state. For Ball Faults, the accuracy reaches 87.5%, with primary misclassifications occurring as Inner Race Faults (4.2%) and Outer Race Faults (8.3%). The accuracy for Inner Race Faults is also 87.5%, dominated by misclassification as Outer Race Faults (12.5%). However, the accuracy for Outer Race Faults is significantly lower at 62.5%, with errors including misclassification as Ball Faults (25.0%) and Inner Race Faults (12.5%). Overall, this method demonstrates substantial room for improvement in diagnosing Outer Race Faults.

In summary, under the 6 dB Gaussian white noise condition, the proposed method achieves significantly higher classification accuracy compared to the other three single-modal approaches, demonstrating its effectiveness on the CWRU dataset. It is noteworthy that for Inner Race Faults, the proposed method exhibits marginally lower accuracy compared to the GADF + VGG16 approach, which may be attributed to feature redundancy and feature conflict introduced during the multimodal fusion process.

(2) Performance under 6 dB pink noise

The experiments were repeated using the dataset added with 6 dB pink noise. The comparison results between the proposed method and other single-modal methods are listed in Table 6.

As can be seen from Table 6, in this noise environment, the proposed method continues to demonstrate outstanding performance, achieving the highest overall accuracy of 96.9%. In contrast, single-modal methods still exhibit instability in such noisy environments. For instance, GADF + VGG16 shows a significant deficiency in diagnosing Ball Faults (79.2%), while RP + VGG16 struggles to accurately identify Outer Race Faults (79.2%). In comparison, the proposed method effectively overcomes these limitations of individual modalities, maintaining an accuracy rate above 95% across all fault categories. These results further validate that the proposed model retains reliable diagnostic capability even when the spectral characteristics of the noise change. Consistent with the previous experiment, these variations in performance are solely attributed to the feature extraction capabilities of the different methods, as the dataset remains strictly balanced.

**Figure 11:**
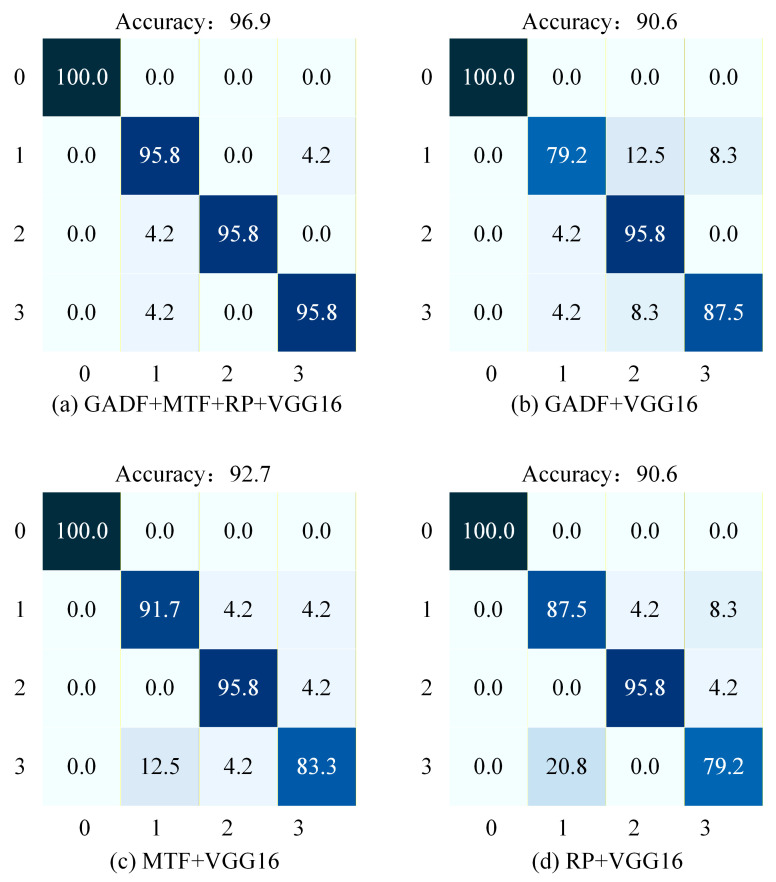
Confusion matrices of the four methods under 6 dB pink noise. (**a**) Proposed GMRVGG (Tri-modal). (**b**) GADF + VGG16. (**c**) MTF + VGG16. (**d**) RP + VGG16. The color intensity represents the percentage of classification accuracy, with darker shades indicating higher values.

To further visualize the detailed classification performance under the impact of pink noise, the confusion matrix of the proposed method is presented in Figure 11. Consistent with the previous setup, the horizontal axis represents the predicted labels, and the vertical axis represents the true labels. The class mapping remains identical to the white noise experiment, where 0, 1, 2, and 3 correspond to Normal condition, Ball Fault, Inner Race Fault, and Outer Race Fault, respectively.

As illustrated in Figure 11a, under the pink noise condition, the proposed GMRVGG16 model achieves an outstanding overall accuracy of 96.9%, demonstrating exceptional robustness. Specifically, the classification accuracy for the Normal state reaches 100% with no misclassifications. For Ball, Inner Race, and Outer Race faults, the model achieves a consistent accuracy of 95.8% for each category. The errors are minimal and evenly distributed: Ball Faults are slightly misclassified as Outer Race Faults (4.2%), Inner Race Faults as Ball Faults (4.2%), and Outer Race Faults as Inner Race Faults (4.2%). Overall, the GMRVGG16 model maintains the highest diagnostic accuracy and stability among all methods.

As illustrated in Figure 11b, the GADF + VGG16 model achieves an overall accuracy of 90.6%. While identifying the Normal state (100.0%) and Inner Race Faults (95.8%) with high precision, it still exhibits a distinct bottleneck in diagnosing Ball Faults. The accuracy for Ball Faults is limited to 79.2%, with primary misclassifications occurring as Inner Race Faults (12.5%) and Outer Race Faults (8.3%). Additionally, Outer Race Faults achieve an accuracy of 87.5%, with minor confusions. This indicates that while the overall performance improves under pink noise compared to white noise, the GADF modality remains less sensitive to Ball Fault features.

As illustrated in Figure 11c, the MTF + VGG16 model achieves an overall accuracy of 92.7%. The classification accuracy for the Normal state reaches 100%. The model performs well on Inner Race Faults (95.8%) and Ball Faults (91.7%). However, the accuracy for Outer Race Faults is relatively lower at 83.3%, with the main error being misclassification as Ball Faults (12.5%). This suggests that under pink noise interference, the MTF modality faces certain challenges in distinguishing between Outer Race and Ball fault patterns.

As illustrated in Figure 11d, the RP + VGG16 model achieves an overall accuracy of 90.6%. Specifically, it attains 100.0% accuracy in classifying the Normal state and 95.8% for Inner Race Faults. However, the model exhibits significant instability in diagnosing Outer Race Faults, where the accuracy drops to 79.2%. The primary source of error is a substantial misclassification of Outer Race Faults as Ball Faults (20.8%). Meanwhile, Ball Faults are identified with 87.5% accuracy. Overall, this method demonstrates a specific deficiency in extracting discriminative features for Outer Race Faults in the presence of pink noise.

In summary, under the 6 dB pink noise condition, the proposed method achieves significantly higher classification accuracy compared to the other three single-modal approaches. By effectively fusing the features from three modalities, the proposed model compensates for the specific shortcomings of individual modalities in identifying certain fault types, thereby demonstrating superior robustness against low-frequency noise interference.

### 4.2. Experiment II: Diagnostic Performance Analysis on Ottawa Dataset

#### 4.2.1. Dataset Introduction

To further validate the generalization capability of the proposed method, the bearing fault diagnosis dataset from the University of Ottawa is employed for experimental validation. This dataset was developed by the Department of Mechanical Engineering at the University of Ottawa. The experimental test rig is configured with a single-phase motor that drives the shaft and a cantilever beam mechanism for applying radial loads [37]. The experiments utilized FAFNIR 203KD ball bearings (Fafnir Bearing Company, New Britain, CT, USA), which are also standard metallic bearings.

Distinct from the CWRU dataset where faults are artificially induced by EDM, the faults in this dataset are generated through accelerated deterioration experiments. By removing the seals and degreasing the drive-end bearings, the bearings are run from a healthy state to failure, thereby simulating the natural evolution of bearing faults. The motor operates at a constant rotational speed of 1750 RPM. The load conditions are maintained at 400 N for inner race faults, outer race faults and cage faults, while ball faults are generated under a no-load condition to facilitate natural fault progression. The dataset covers different health conditions, categorized as healthy, developing faults, and faulty states. The bearing fault signals are sampled at 42,000 Hz.

For the experiments in this section, vibration signals acquired at a sampling frequency of 42,000 Hz were selected. Inner Race faults, Outer Race faults, and Cage faults were chosen. It is important to note that Ball faults were excluded from this experiment to ensure rigorous control of variables. According to the dataset specifications, Ball faults were generated under a no-load condition, whereas all other health states (Normal, Inner Race, Outer Race, and Cage) were recorded under a consistent nominal load of 400 N. Excluding Ball faults eliminates load variation as a confounding factor, ensuring that the classification is based solely on fault characteristics. For each selected fault type, signals corresponding to both ‘developing fault’ and ‘faulty’ states were selected and combined with signals from the normal state, resulting in a total of seven distinct categories of bearing fault signal data. Consistent with the previous categorization strategy, signals of the same fault type but representing different severity levels are grouped under the same label, resulting in four final categories: ‘Normal’, ‘IR’, ‘OR’, and ‘Cage’.

**Figure 12:**
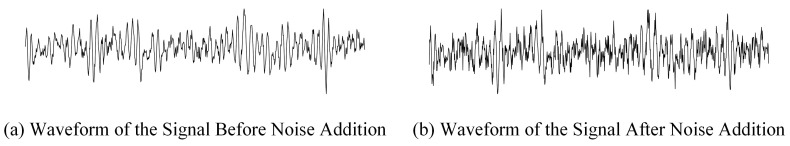
Signal waveform comparison under 6 dB Gaussian white noise. (**a**) Original signal before noise addition. (**b**) Signal after adding 6 dB Gaussian white noise.

To ensure a consistent and rigorous evaluation of the model’s generalization ability, the noise injection protocol employed in Section 4.1 was strictly replicated for the Ottawa dataset. Consistent with the previous experiment, both 6 dB Gaussian white noise and 6 dB pink noise were individually introduced into the raw signals prior to sample segmentation. A comparison of the signal waveforms before and after adding 6 dB Gaussian white noise is shown in Figure 12.

**Figure 13:**
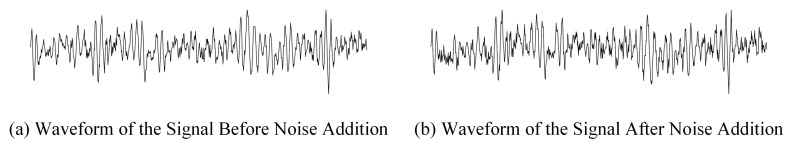
Signal waveform comparison under 6 dB pink noise. (**a**) Original signal before noise addition. (**b**) Signal after adding 6 dB pink noise.

As shown in Figure 12, the signal waveform undergoes a significant transformation, similar to that observed in the CWRU dataset. The introduction of 6 dB Gaussian white noise leads to a pronounced increase in random irregularities and substantial fluctuations in amplitude, validating the successful simulation of a harsh noisy environment. A comparison of the signal waveforms before and after adding 6 dB pink noise is shown in Figure 13.

As shown in Figure 13, the waveform of the signal also exhibits significant changes after the addition of 6 dB pink noise.

To prevent data leakage and ensure experimental consistency, the data partitioning strategy employed in Section 4.1 was strictly followed. For each noise scenario, the data points were partitioned into training and test sets in an 8:2 ratio. Subsequently, samples were obtained through sequential segmentation with a sample length of 512 data points and an overlap ratio of 50%. The details of the processed Ottawa dataset partition are listed in Table 7.

As shown in Table 7, the dataset is categorized into four classes (Normal, IR, OR, Cage) labeled 0 to 3. Consistent with the data balancing strategy employed in Section 4.1, a strictly balanced dataset was constructed for this experiment as well. For each noise scenario, every category comprises 108 training samples and 24 test samples, ensuring a uniform distribution for fair model evaluation.

#### 4.2.2. Experimental Results

(1) Performance under 6 dB Gaussian white noise

For the method proposed in this paper, the experimental results on the Ottawa dataset under the 6 dB Gaussian white noise condition are listed in Table 8.

As can be seen from Table 8, in this noise environment, the proposed method achieves significantly higher accuracy than other single-modal methods, reaching an overall accuracy of 95.8%. Under the interference of 6 dB Gaussian white noise, single-modal methods exhibit distinct vulnerabilities in identifying specific fault types. For instance, the MTF + VGG16 model shows a severe deficiency in diagnosing Inner Race Faults, with an accuracy of only 58.3%. Similarly, RP + VGG16 struggles to distinguish Inner Race Faults (70.8%) and Cage Faults (83.3%). In contrast, the tri-modal fusion approach effectively compensates for these single-modal limitations, achieving 100% accuracy in recognizing Normal, Outer Race Faults, and Cage Faults, and significantly improving the recognition rate of Inner Race Faults to 83.3%. Consistent with the previous experiment, these variations in performance are solely attributed to the feature extraction capabilities of the different methods, as the dataset remains strictly balanced.

**Figure 14:**
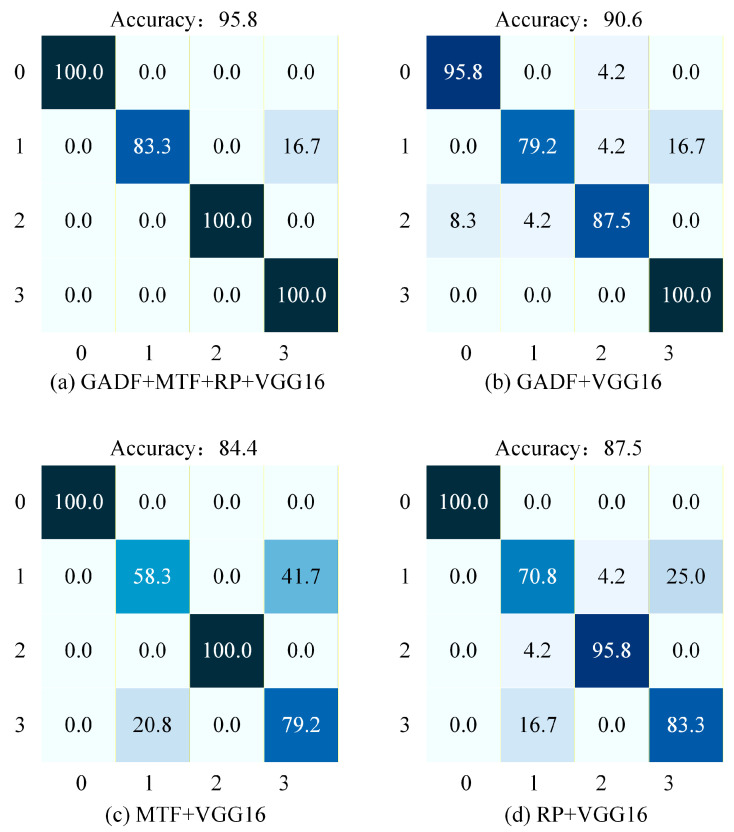
Confusion matrices of the four methods under 6 dB Gaussian white noise. (**a**) Proposed GMRVGG (Tri-modal). (**b**) GADF + VGG16. (**c**) MTF + VGG16. (**d**) RP + VGG16. The color intensity represents the percentage of classification accuracy, with darker shades indicating higher values.

To intuitively assess the classification performance on the Ottawa dataset under 6 dB Gaussian white noise, the confusion matrices for the four methods are displayed in Figure 14. In these matrices, the horizontal axis represents the predicted labels, while the vertical axis represents the true labels, where 0 represents the Normal condition, 1 represents the Inner Race Fault, 2 represents the Outer Race Fault, and 3 represents the Cage Fault.

As illustrated in Figure 14a, the proposed GMRVGG16 model achieves a superior overall accuracy of 95.8% and demonstrates exceptional stability. Specifically, the classification accuracy for the Normal state, Outer Race Faults, and Cage Faults all reach a perfect 100% with no misclassifications. The only minor confusion occurs in identifying Inner Race Faults, where the accuracy is 83.3%, with the remaining samples being misclassified as Cage Faults (16.7%). Overall, the proposed model effectively captures the discriminative features of different faults, minimizing confusion even in the presence of white noise.

As illustrated in Figure 14b, the GADF + VGG16 model achieves an overall accuracy of 90.6%. It attains 100% accuracy in classifying Cage Faults and 95.8% for the Normal state. However, its performance drops for Inner Race Faults (79.2%), with misclassifications occurring as Cage Faults (16.7%) and Outer Race Faults (4.2%). Similarly, for Outer Race Faults, the accuracy is 87.5%, with some confusion with Normal states (8.3%). Overall, while GADF performs well on Cage faults, it exhibits limitations in accurately distinguishing Inner Race faults.

As illustrated in Figure 14c, the MTF + VGG16 model achieves the lowest overall accuracy of 84.4%, revealing a severe deficiency in distinguishing specific fault types. While it perfectly identifies Normal and Outer Race conditions (100%), it fails significantly in diagnosing Inner Race Faults, achieving an accuracy of only 58.3%. A substantial portion of Inner Race samples (41.7%) are misclassified as Cage Faults. Conversely, Cage Faults (79.2%) are also frequently misclassified as Inner Race Faults (20.8%). This indicates that the MTF modality struggles to extract unique features to separate Inner Race and Cage Faults under white noise interference.

As illustrated in Figure 14d, the RP + VGG16 model achieves an overall accuracy of 87.5%, showing a similar pattern of confusion. Specifically, it attains 100.0% accuracy for the Normal state and 95.8% for Outer Race Faults. However, the accuracy for Inner Race Faults is low at 70.8%, dominated by misclassification as Cage Faults (25.0%). For Cage Faults, the accuracy is 83.3%, with primary errors being misclassified as Inner Race Faults (16.7%). Overall, this method demonstrates a specific weakness in distinguishing between Inner Race and Cage faults.

In summary, under the 6 dB Gaussian white noise condition, the proposed method achieves significantly higher classification accuracy compared to the other three single-modal approaches, demonstrating its effectiveness on the Ottawa dataset.

(2) Performance under 6 dB pink noise

The experiments were repeated using the dataset added with 6 dB pink noise. The comparison results between the proposed method and other single-modal methods are listed in Table 9.

As can be seen from Table 9, in this noise environment, the proposed method continues to demonstrate outstanding performance, achieving the highest overall accuracy of 95.8%. In contrast, single-modal methods still exhibit specific vulnerabilities when facing pink noise interference. For instance, the RP + VGG16 model struggles to accurately identify Inner Race Faults, with an accuracy of only 75.0%. Similarly, the MTF + VGG16 model shows limited capability in distinguishing Inner Race Faults (79.2%). While the GADF + VGG16 model achieves perfect recognition for Cage Faults, its performance on Outer Race Faults (87.5%) remains suboptimal. In comparison, the proposed tri-modal fusion approach effectively integrates the strengths of individual features to overcome these limitations.

Consistent with previous experiments, these performance differences are solely attributed to the distinct feature representation capabilities of each method, as the dataset remains strictly balanced. The present experimental results further confirm that the proposed method possesses strong generalization capability, maintaining high reliability even under different noise patterns and mechanical systems.

**Figure 15:**
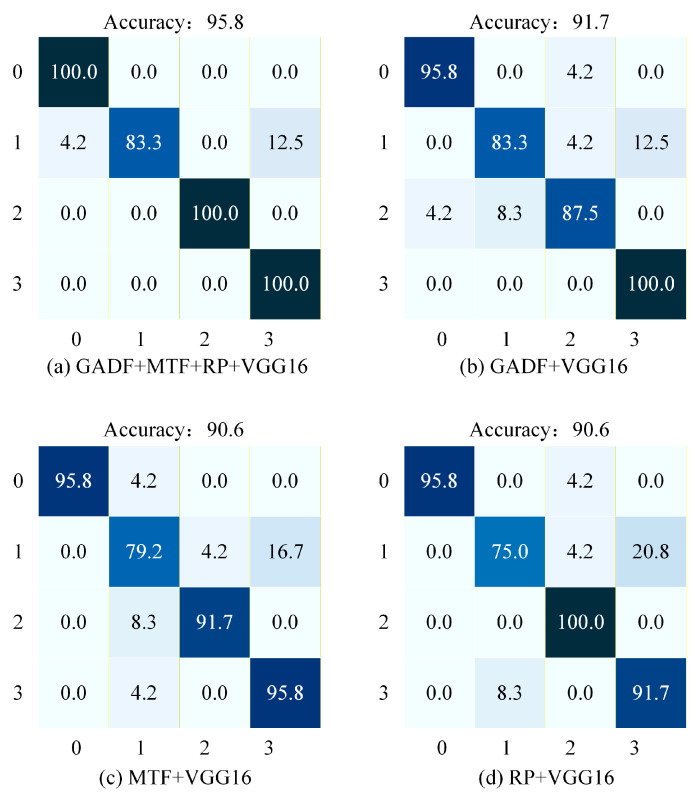
Confusion matrices of the four methods under 6 dB pink noise. (**a**) Proposed GMRVGG (Tri-modal). (**b**) GADF + VGG16. (**c**) MTF + VGG16. (**d**) RP + VGG16. The color intensity represents the percentage of classification accuracy, with darker shades indicating higher values.

To further visualize the detailed classification performance on the Ottawa dataset under the impact of pink noise, the confusion matrices for the four methods are displayed in Figure 15. Consistent with the previous setup, the horizontal axis represents the predicted labels, and the vertical axis represents the true labels. The class mapping remains identical to the white noise experiment, where 0, 1, 2, and 3 correspond to Normal condition, Inner Race Fault, Outer Race Fault, and Cage Fault, respectively.

As illustrated in Figure 15a, the proposed GMRVGG16 model achieves a superior overall accuracy of 95.8% and demonstrates exceptional stability under the impact of pink noise. Specifically, the classification accuracy for the Normal state, Outer Race Faults, and Cage Faults all reach a perfect 100% with no misclassifications. The primary source of error is confined to Inner Race Faults, where the accuracy is 83.3%. These misclassified samples are attributed to Normal states (4.2%) and Cage Faults (12.5%). Overall, despite the slight confusion in Inner Race patterns, the proposed model effectively integrates the strengths of multiple modalities to minimize errors, maintaining the highest diagnostic performance among all methods.

As illustrated in Figure 15b, the GADF + VGG16 model achieves an overall accuracy of 91.7%. It attains 100.0% accuracy in classifying Cage Faults, demonstrating high sensitivity to this fault type. However, its performance exhibits limitations in distinguishing other fault categories. The accuracy for Inner Race Faults is 83.3%, with misclassifications occurring as Outer Race Faults (4.2%) and Cage Faults (12.5%). Similarly, for Outer Race Faults, the accuracy is 87.5%, with confusion primarily involving Inner Race Faults (8.3%). This indicates that while the GADF modality is robust for Cage faults, it struggles to fully separate the features of Inner and Outer Race faults in a pink noise environment.

As illustrated in Figure 15c, the MTF + VGG16 model achieves an overall accuracy of 90.6%. While it maintains high precision for Normal states (95.8%) and Cage Faults (95.8%), it reveals a distinct deficiency in diagnosing Inner Race Faults. The accuracy for Inner Race Faults is limited to 79.2%, with a significant portion of samples being misclassified as Cage Faults (16.7%). Additionally, Outer Race Faults achieve an accuracy of 91.7%, with minor misclassification as Inner Race Faults (8.3%). This suggests that under pink noise interference, the MTF modality faces challenges in extracting unique features to distinguish Inner Race Faults from Cage Faults.

As illustrated in Figure 15d, the RP + VGG16 model achieves an overall accuracy of 90.6%, exhibiting a polarized performance distribution. It attains perfect classification (100.0%) for Outer Race Faults, outperforming the GADF and MTF models in this category. However, it demonstrates the lowest performance for Inner Race Faults, where the accuracy drops to 75.0%. The primary error is a substantial misclassification of Inner Race Faults as Cage Faults (20.8%). Overall, while the RP modality is highly effective for Outer Race features, it shows a specific weakness in capturing the discriminative patterns of Inner Race faults.

In summary, under the 6 dB pink noise condition, the proposed method achieves significantly higher classification accuracy compared to the other three single-modal approaches. These results further demonstrate the exceptional generalization capability and robustness of the proposed method on the Ottawa dataset.

### 4.3. Comprehensive Analysis and Evaluation

#### 4.3.1. Ablation Study

To further investigate the specific contribution of each branch within the proposed tri-modal architecture and explicitly rule out the possibility of feature redundancy, an ablation study was conducted. Three dual-modal fusion fault diagnosis models were constructed by sequentially removing the RP, MTF, and GADF branches from the original three-branch model. To ensure consistency with the initial baseline performance analysis, these experiments were conducted on the CWRU dataset augmented with 6 dB Gaussian white noise. To maintain narrative conciseness and avoid redundant data presentation across all four distinct dataset and noise scenarios, these experiments were specifically conducted on the CWRU dataset augmented with 6 dB Gaussian white noise. This condition was selected as a representative benchmark to effectively demonstrate the structural necessity of the fusion strategy. The experimental results are listed in Table 10.

**Figure 16:**
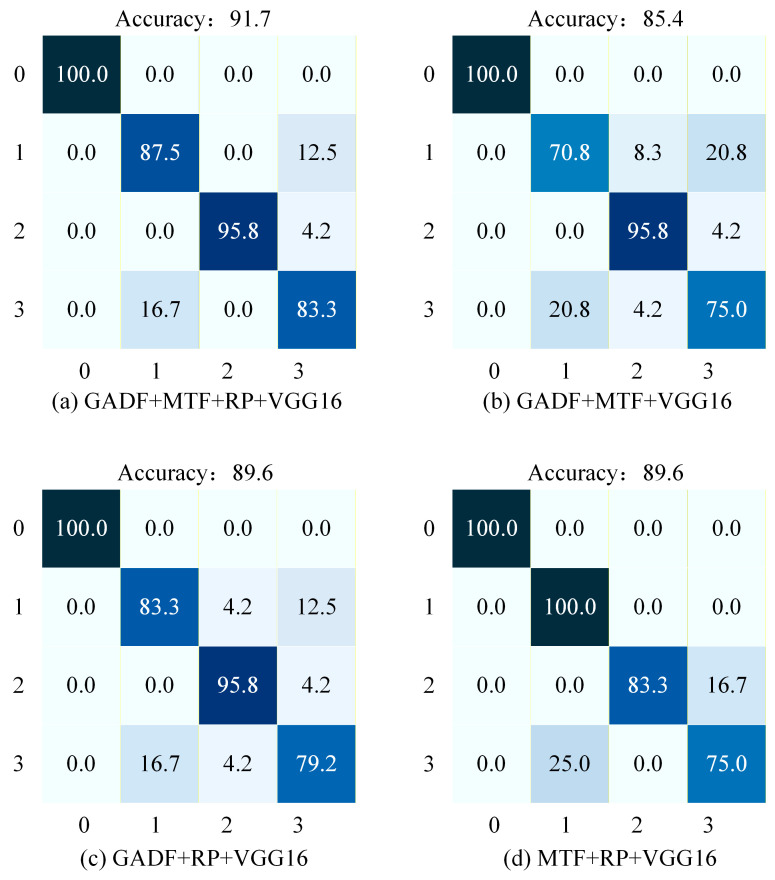
Confusion matrix for the ablation study. (**a**) Proposed GMRVGG (Tri-modal). (**b**) GADF + MTF + VGG16. (**c**) GADF + RP + VGG16. (**d**) MTF + RP + VGG16. The color intensity represents the percentage of classification accuracy, with darker shades indicating higher values.

As shown in Table 10, the proposed method achieves higher accuracy than the three dual-modal fusion methods. Among the three dual-modal fusion methods, both GADF + RP + VGG16 and MTF + RP + VGG16 achieved the highest accuracy of 89.6%. However, the proposed tri-modal fusion method still outperforms these dual-modal approaches. Similarly, to more intuitively evaluate the model’s performance, a confusion matrix was introduced to observe the effectiveness of fault classification. Figure 16 shows the confusion matrix from the ablation study. In these matrices, the horizontal axis represents the predicted labels, while the vertical axis represents the true labels, where 0 represents the Normal condition, 1 represents the Ball Fault, 2 represents the Inner Race Fault, and 3 represents the Outer Race Fault.

As illustrated in Figure 16b, the GADF + MTF + VGG16 model achieves an overall accuracy of 85.4%. Specifically, it attains 100.0% accuracy in classifying the Normal state. However, the accuracy for Ball Faults is relatively low at 70.8%, with primary misclassifications occurring as Inner Race Faults (8.3%) and Outer Race Faults (20.8%). For Inner Race Faults, the accuracy reaches 95.8%, dominated by misclassification as Outer Race Faults (4.2%). The accuracy for Outer Race Faults is 75.0%, with errors including misclassification as Ball Faults (20.8%) and Inner Race Faults (4.2%).

As illustrated in Figure 16c, the GADF + RP + VGG16 model achieves an overall accuracy of 89.6%. It demonstrates perfect classification (100.0% accuracy) for the Normal state. For Ball Faults, the accuracy is 83.3%, with primary misclassifications occurring as Inner Race Faults (4.2%) and Outer Race Faults (12.5%). The accuracy for Inner Race Faults reaches 95.8%, dominated by misclassification as Outer Race Faults (4.2%). However, the accuracy for Outer Race Faults is 79.2%, with main errors including misclassification as Ball Faults (16.7%) and Inner Race Faults (4.2%).

As illustrated in Figure 16d, the MTF + RP + VGG16 model also achieves an overall accuracy of 89.6%. It demonstrates perfect classification (100.0% accuracy) for both the Normal state and Ball Faults. However, the accuracy for Outer Race Faults is the lowest at 75.0%, with the primary error being misclassification as Ball Faults (25.0%). For Inner Race Faults, the accuracy is 83.3%, dominated by misclassification as Outer Race Faults (16.7%).

Overall, both the GADF + RP + VGG16 and MTF + RP + VGG16 models demonstrate relatively good performance; however, the overall accuracy of the proposed method remains higher than these two approaches. It is noteworthy that for Ball Faults, the proposed method exhibits lower accuracy compared to the MTF + RP + VGG16 model. This observation indicates that the feature vectors extracted from GADF images, when fused, exert a certain adverse impact on the model’s diagnostic performance for this specific fault type. To deeply investigate the cause of this specific inter-modality conflict and to mathematically prove the overall necessity of fusing these three distinct modalities, a quantitative feature similarity analysis is conducted in the following Section 4.3.2.

In summary, this ablation study systematically validates the contribution of each branch within the multimodal fusion architecture by comparing the performance of the proposed complete model against three dual-modal simplified models (constructed by sequentially removing the RP, MTF, or GADF branches). The experimental data confirms that the tri-modal fusion strategy significantly enhances the comprehensive performance of fault diagnosis. With the complete model achieving an overall accuracy of 91.7%, it outperforms all dual-modal combinations, justifying the necessity of the proposed tri-modal framework.

Additionally, a brief discussion on interpretability is provided here. It is acknowledged that the feature vectors extracted by the CNN backbone are high-level abstract representations generated through complex nonlinear mappings. However, their physical interpretability can be inferred from the encoding mechanisms of the input modalities and is further validated by the results of ablation studies. Specifically, since the GADF mathematically maps the 1D signal to a 2D matrix based on the trigonometric difference between each pair of time points, the features extracted from this branch inherit information about the temporal correlations and amplitude fluctuations of the signal. Similarly, as the MTF images are constructed from transition probabilities, the corresponding features effectively encode the dynamic statistical properties of the system, reflecting the probabilities of transitioning between different energy states. Furthermore, given that the RP visualizes the phase space trajectories, the extracted features primarily characterize the periodicity and non-stationarity of the fault impulses. This interpretation is further supported by the results in Table 10, where removing any single branch leads to a noticeable decline in accuracy. This implies that the specific physical information provided by each modality has been successfully captured by the network and plays a unique, non-redundant role in the final fault diagnosis.

#### 4.3.2. Feature Redundancy and Modality Conflict Analysis

**Figure 17:**
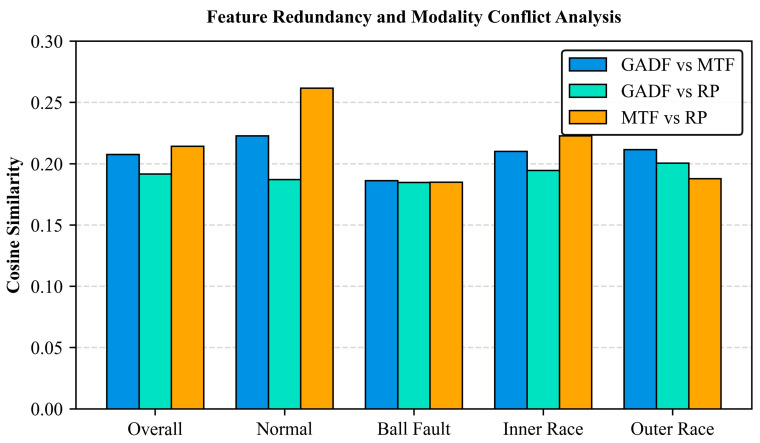
Quantitative analysis of feature similarity among the GADF, MTF, and RP branches across different health conditions. The consistently low cosine similarity indicates strong complementarity.

To quantify the inter-modality relationships and investigate the performance anomaly observed during the Ball Fault diagnosis (Section 4.3.1), we calculated the cosine similarity among the 4096-dimensional feature vectors extracted by the GADF, MTF, and RP branches. Consistent with the ablation study, this analysis was conducted using the CWRU dataset augmented with 6 dB Gaussian white noise. The results are illustrated in Figure 17.

As depicted in Figure 17, the average cosine similarities among the three modalities are consistently low, hovering around 0.20. In high-dimensional spaces, such near-zero values indicate that the extracted features are largely orthogonal. This suggests that the GADF, MTF, and RP branches successfully capture distinct, non-overlapping physical properties of the signals. This lack of feature redundancy confirms their strong complementarity, providing a clear rationale for the tri-modal fusion strategy.

Notably, the similarity drops to its lowest level under the Ball Fault condition. This phenomenon helps explain the slight performance degradation of the GADF-related combinations observed in the ablation study. Since Ball Fault impulses are inherently weak and easily masked by the severe 6 dB background noise, the GADF encoding—which computes trigonometric differences between temporal points—becomes highly sensitive to high-frequency random interference. Consequently, the GADF feature space misaligns with those of MTF and RP, leading to a geometric deviation or “modality conflict.” While this conflict introduces minor interference during fusion, the overall tri-modal framework effectively neutralizes it. By integrating the robust topological dynamics from RP and the stable statistical transitions from MTF, the network successfully compensates for GADF’s vulnerability under extreme noise.

#### 4.3.3. Comparison with State-of-the-Art Methods

To further evaluate the comprehensive performance and environmental robustness of the proposed GMRVGG, it was compared against three recent advanced 1D deep learning methods: 1D-ResNet, Attention-based BiTCN (Attn-BiTCN), and Multi-Scale Dilated Convolution and Dense Temporal Convolutional Network (MSDC-DenseTCN). To ensure a fair comparison, all baseline models were trained and tested under the exact same configurations across four distinct diagnostic scenarios, encompassing two datasets (CWRU and Ottawa) and two severe noise conditions (6 dB White noise and Pink noise). The diagnostic accuracies are summarized in Table 11.

As shown in Table 11, the 1D baseline models exhibit significant sensitivity to the type of background noise, revealing a critical cross-domain vulnerability. For instance, while Attn-BiTCN achieves a 100% accuracy on the Ottawa dataset under white noise, its performance drastically drops to 68.75% when applied to the CWRU dataset under low-frequency pink noise. This severe fluctuation indicates that traditional 1D temporal models tend to overfit to specific frequency distributions and struggle to maintain robustness when the spectral characteristics of the interference change.

In contrast, GMRVGG maintains a more consistent accuracy range of 91.7% to 96.9% across the four evaluated scenarios. The results indicate that mapping 1D signals into multiple 2D feature spaces (GADF, MTF, and RP) can reduce the impact of spectral variations in background noise. While the 1D state-of-the-art models also achieve competitive performance, the proposed method demonstrates more consistent stability under the evaluated experimental conditions. These findings suggest that the proposed framework offers a viable alternative for fault diagnosis in environments with varying interference.

However, compared to lightweight 1D methods, the proposed method requires higher computational overhead due to the parallel execution of three VGG16 backbones. Therefore, direct deployment on memory-limited portable devices is challenging. To enable edge deployment, exploring model compression techniques will be a necessary direction for future research.

## 5. Conclusions

To address the limitations of existing bearing fault diagnosis methods, which often rely on single-modal data or a solitary signal-to-image conversion technique—leading to insufficient information dimensionality and inadequate feature representation—this paper proposes a bearing fault diagnosis method (GMRVGG) based on tri-modal image feature fusion. First, in order to rigorously simulate complex real-world industrial environments, severe 6 dB Gaussian white noise and 6 dB pink noise were injected into the raw signals prior to sample partitioning. Subsequently, the 1D vibration signals are converted into 2D images by employing three distinct signal-to-image transformation methods: GADF, MTF, and RP. Shallow to deep features are extracted from these images using the VGG16 architecture, and the resulting feature vectors are fused via concatenation. Finally, fault diagnosis is accomplished by a fully connected classifier. Extensive experiments conducted on both the Case Western Reserve University (CWRU) and the University of Ottawa bearing datasets demonstrate the exceptional robustness of the proposed method. It achieved overall accuracies of up to 96.9% and 95.8% on the two datasets respectively, consistently outperforming traditional single-modal approaches across different noise interferences. This effectively addresses the issues of insufficient information dimensionality and inadequate feature representation, providing a highly reliable and generalizable solution for intelligent bearing condition monitoring.

Nevertheless, the current evaluation is based on constant load and speed conditions, which still differs from actual industrial datasets. To further bridge the gap between laboratory research and complex industrial applications, our future work will focus on addressing non-stationary operating conditions and cross-sensor variability. Specifically, inspired by the advanced digital twin-guided physical-virtual denoising framework [38] and the innovative concept of positive-incentive noise [39], we plan to explore these directions by integrating domain adaptation techniques to further enhance diagnostic intelligence and reliability under extreme industrial conditions.

Furthermore, to ensure the long-term adaptability and computational efficiency of the model in continuous operations, we will also explore incremental fault diagnosis methodologies [40]. Integrating lifelong learning strategies with dynamic learnable pruning mechanisms [41] will be a crucial next step to evolve our current static network into a lightweight, continuously adaptive framework capable of learning emerging fault patterns without catastrophic forgetting.

## Figures and Tables

**Table 1:** Network Parameters of the VGG16 Feature Extractor.

Layer	Output Size	Kernel Size	Stride	Repeat	Output Channels
Input	224 × 224 × 3	-	-	-	3
Conv1 + ReLU	224 × 224	3 × 3	1	2	64
MaxPool1	112 × 112	2 × 2	2	1	-
Conv2 + ReLU	112 × 112	3 × 3	1	2	128
MaxPool2	56 × 56	2 × 2	2	1	-
Conv3 + ReLU	56 × 56	3 × 3	1	3	256
MaxPool3	28 × 28	2 × 2	2	1	-
Conv4 + ReLU	28 × 28	3 × 3	1	3	512
MaxPool4	14 × 14	2 × 2	2	1	-
Conv5 + ReLU	14 × 14	3 × 3	1	3	512
MaxPool5	7 × 7	2 × 2	2	1	-
AvgPool	7 × 7	-	-	1	512
Flatten	25,088	-	-	1	-
Linear	4096	-	-	1	4096

**Table 2:** Network Parameters of the Fully Connected Classifier.

Layer	Output Size	Output Channels
Input	12,288	12,288
Linear1 + BN1 + ReLU1 + Dropout1	1024	1024
Linear2 + BN2 + ReLU2 + Dropout2	512	512
Output (Linear3)	4	4

**Table 3:** Hyperparameters for Model.

Parameter	Feature Extractor	Fully Connected Classifier
Batch Size	2	108/24
Optimizer	Adam	Adam
Initial Learning Rate	1 × 10^−4^	1 × 10^−4^
LR Scheduler	StepLR (step = 40, gamma = 0.97)	StepLR (step = 40, gamma = 0.95)
Weight Decay	-	1 × 10^−4^
Epochs	50	50
Loss Function	Cross-Entropy Loss	Cross-Entropy Loss

**Table 4:** Processed CWRU Dataset.

Fault	Label	Dataset (Train/Test)
Ball	1	108/24
IR	2	108/24
OR	3	108/24
Normal	0	108/24

**Table 5:** Method comparison under 6 dB Gaussian white noise.

Method	Ball	IR	OR	Normal	Overall Accuracy
GADF + VGG16	58.3%	100.0%	70.8%	100.0%	82.3%
MTF + VGG16	79.2%	79.2%	62.5%	100.0%	80.2%
RP + VGG16	87.5%	87.5%	62.5%	100.0%	84.4%
Proposed method	87.5%	95.8%	83.3%	100.0%	91.7%

**Table 6:** Method comparison under 6 dB pink noise.

Method	Ball	IR	OR	Normal	Overall Accuracy
GADF + VGG16	79.2%	95.8%	87.5%	100.0%	90.6%
MTF + VGG16	91.7%	95.8%	83.3%	100.0%	92.7%
RP + VGG16	87.5%	95.8%	79.2%	100.0%	90.6%
Proposed method	95.8%	95.8%	95.8%	100.0%	96.9%

**Table 7:** Processed Ottawa Dataset.

Fault	Label	Dataset (Train/Test)
IR	1	108/24
OR	2	108/24
Cage	3	108/24
Normal	0	108/24

**Table 8:** Method comparison under 6 dB Gaussian white noise.

Method	IR	OR	Cage	Normal	Overall Accuracy
GADF + VGG16	79.2%	87.5%	100.0%	95.8%	90.6%
MTF + VGG16	58.3%	100.0%	79.2%	100.0%	84.4%
RP + VGG16	70.8%	95.8%	83.3%	100.0%	87.5%
Proposed method	83.3%	100.0%	100.0%	100.0%	95.8%

**Table 9:** Method comparison under 6 dB pink noise.

Method	IR	OR	Cage	Normal	Overall Accuracy
GADF + VGG16	83.3%	87.5%	100.0%	95.8%	91.7%
MTF + VGG16	79.2%	91.7%	95.8%	95.8%	90.6%
RP + VGG16	75.0%	100.0%	91.7%	95.8%	90.6%
Proposed method	83.3%	100.0%	100.0%	100.0%	95.8%

**Table 10:** Ablation Study Results.

Method	Ball	IR	OR	Normal	Overall Accuracy
GADF + MTF + VGG16	70.8%	95.8%	75.0%	100.0%	85.4%
GADF + RP + VGG16	83.3%	95.8%	79.2%	100.0%	89.6%
MTF + RP + VGG16	100.0%	83.3%	75.0%	100.0%	89.6%
Proposed method	87.5%	95.8%	83.3%	100.0%	91.7%

**Table 11:** Comparative Experimental Results.

Method	CWRU	CWRU	Ottawa	Ottawa
Noisy Environment	6 dB White Noise	6 dB Pink Noise	6 dB White Noise	6 dB Pink Noise
1D-ResNet	47.9%	39.6%	47.9%	50.0%
Attn-BiTCN	88.5%	68.8%	100.0%	94.8%
MSDC-DenseTCN	65.6%	75.6%	100.0%	85.42%
Proposed method	91.7%	96.9%	95.8%	95.8%

## Data Availability

The original data presented in the study are openly available in the Case Western Reserve University Bearing Data Center Website and Mendeley Data at https://engineering.case.edu/bearingdatacenter (accessed on 1 September 2025) and https://data.mendeley.com/datasets/y2px5tg92h/5 (accessed on 1 September 2025).

## Abbreviations

The following abbreviations are used in this manuscript:

1D	One Dimensional
2D	Two Dimensional
Attn-BiTCN	Attention-based Bidirectional Temporal Convolutional Network
BiTCN	Bidirectional Temporal Convolutional Network
BN/BatchNorm	Batch Normalization
CNN	Convolutional Neural Networks
CWRU	Case Western Reserve University
DenseTCN	Dense Temporal Convolutional Network
EDM	Electric Discharge Machining
GADF	Gramian Angular Difference Field
GAF	Gramian Angular Field
GMRVGG	GADF-MTF-RP-VGG16
ILSVRC	ImageNet Large Scale Visual Recognition Challenge
IR	Inner Race
MSDC	Multi-Scale Dilated Convolution
MTF	Markov Transition Field
OR	Outer Race
ReLU	Rectified Linear Unit
ResNet	Residual Networks
RNN	Recurrent Neural Networks
RP	Recurrence Plot
RPM	Revolutions Per Minute
SST	Synchrosqueezed Wavelet Transform
SVMD	Successive Variational Mode Decomposition
VGG	Visual Geometry Group
